# The effect of men who have sex with men (MSM) on the spread of sexually transmitted infections

**DOI:** 10.1186/s12976-021-00148-9

**Published:** 2021-10-11

**Authors:** Hiromu Ito, Taro Yamamoto, Satoru Morita

**Affiliations:** 1 Department of International Health and Medical Anthropology, Institute of Tropical Medicine, Nagasaki University, Nagasaki, Nagasaki 852-8523 Japan; 2 Department of Mathematical and Systems Engineering, Shizuoka University, Hamamatsu, Shizuoka 432-8561 Japan; 3 Department of Environment and Energy Systems, Graduate School of Science and Technology, Shizuoka University, Hamamatsu, Shizuoka 432-8561 Japan

**Keywords:** Sexually transmitted diseases, Type-reproduction number, Complex networks, MSM contact, Bisexual bridge

## Abstract

Sexually transmitted infections (STIs) have remained a worldwide public health threat. It is difficult to control the spread of STIs, not only because of heterogeneous sexual transmission between men and women but also because of the complicated effects of sexual transmission among men who have sex with men (MSM) and mother-to-child transmission. Many studies point to the existence of a ‘bisexual bridge’, where STIs spread from the MSM network via bisexual connections. However, it is unclear how the MSM network affects heterosexual networks as well as mother-to-child transmission. To analyse the effect of MSM on the spread of STIs, we divided the population into four subpopulations: (i) women, (ii) men who have sex with women only (MSW), (iii) men who have sex with both men and women (MSMW), (iv) men who have sex with men exclusively (MSME). We calculated the type-reproduction numbers of these four subpopulations, and our analysis determined what preventive measures may be effective. Our analysis shows the impact of bisexual bridge on the spread of STIs does not outweigh their population size. Since MSM and mother-to-child transmission rates do not have a strong synergistic effect when combined, complementary prevention measures are needed. The methodologies and findings we have provided here will contribute greatly to the future development of public health.

**Supplementary Information:**

The online version contains supplementary material available at 10.1186/s12976-021-00148-9.

## Introduction

The relationship between men who have sex with men (MSM) and sexually transmitted infections (STIs) has been extensively studied because the infection rates of various STIs in MSM are greater than those in women and men who have sex with women only (MSW) [1]. MSM have been widely studied in research on HIV; however, MSM were exposed to many STIs before the spread of HIV [2]. The term MSM has been used since 1990 to reflect the epidemiological belief that behaviour is responsible for the risk of STIs [3]. MSM are defined only by their behaviour, not their sexual identity. Many studies have estimated the proportion of MSM among the male population in various countries [4–12] (Table 1). According to these studies, the proportion of MSM among the male population is approximately 1% to 5% in many countries. MSM should be classified into two subpopulations: men who have sex with men exclusively (MSME) and men who have sex with men and women (MSMW). In the research studies shown in Table 1, MSME and MSMW are not distinguished; thus, the proportions of MSME and MSMW are unknown.

**Table 1:** The proportions of MSM in male populations

Countries and references	Proportion of MSM (%)	Publication year
USA [4]	6.4	2011
USA [5]	1.5–6.0	2016
Canada (Metro Vancouver) [6]	2.9	2018
Japan [7]	2.87	2012
Vietnam [8]	0.68	2019
38 Countries in Europe [9]	0.03–5.6	2013
Brazil [10]	3.5	2019
Dominican Republic [11]	1.2	2018
Côte d’Ivoire [12]	0.5–10	2019

When we consider the persistence of STIs in human society, transmission routes are key factors [13, 14]. Hepatitis B virus (HBV) and human T-cell leukaemia virus type I (HTLV-1) are endemic STIs that are spread by sexual (horizontal) transmission and mother-to-child (vertical) transmission. What makes these viruses so persistent is that vertically infected infants can develop to reproductive age and spread the infections through sexual transmission [15–18]. We suspect that the presence of STIs, which have robust intragenerational routes of transmission, combined with mother-to-child transmission, which allows transmission across generations, have contributed significantly to the persistence of these viruses. If we can mathematically estimate the spreading efficiency of each of these transmission routes, we can understand persistence strategies in the context of the ecological and evolutionary biology of STIs. This will also help us develop vaccination strategies for HBV, for which a safe vaccine exists. To address the question of whether an STI can persist, we need to take a long-term view, and it is necessary to consider not only transmission due to MSM but also mother-to-child transmission.

There are many mathematical models of STIs focusing on human sexual networks [19, 20]. It is well known that human sexual networks are heterogeneous; most people have sexual contact with only a few partners, but a small number of sexually active people have sexual contact with hundreds of partners. This heterogeneity is believed to contribute to the persistence of STIs [21]. In a simple epidemic model of a network that does not consider sex [22], the basic reproduction number ${R}_{0}$, which measures the number of infections produced by an infected individual on average, is determined by the fluctuation in sexual activity $a$:

1 $$C=\frac{\langle {a}^{2}\rangle }{\langle a\rangle }.$$

Here, $a$ is the degree of sexual activity, which is proportional to the number of sexual contacts, and thus, $C$ is the variation of $a$ [19]. If we consider heterosexual contacts between men and women (i.e., the network is bipartite), ${R}_{0}$ is proportional to the geometric mean of the value $C$ for men and women [19]. If the contact frequency distribution is a power distribution and the network is scale-free [23, 24], then ${R}_{0}$ and $C$ can approach infinity. Many studies have noted that sexual contact networks are scale-free [25–28].

There are few models that consider the following two points simultaneously. The first point is mother-to-child (vertical) transmission. Mother-to-child transmission (e.g., via the placenta, birth blood exposure, breast feeding) is an important transmission route of STIs [14, 29, 30]. Therefore, in our previous studies, we built models that simultaneously considered sexual (horizontal) and mother-to-child (vertical) transmission under the scale-free property of sexual contact frequency [31, 32]. In particular, a realistic model presented in our latest work took into account adult and infant mortality from infection, infertility and stillbirth caused by infection, as well as recovery with treatment [32]. Although many STIs can cause serious harm to infants infected via mother-to-child transmission, HBV and HTLV-1 do not immediately adversely affect infected infants, and infected infants can grow and spread the infection [33, 34].

The second point is how the MSM network indirectly affects mother-to-child transmission through heterosexual networks via bisexual connections, which are called ‘bisexual bridges’ [35]. Bisexual and homosexual contacts play an important role in the spread of STIs [36]. Fernando argued that the current Centers for Disease Control (CDC) risk subpopulation classification, in which MSMW and MSME are included in a single MSM subpopulation, makes it impossible to know the extent of STI (e.g., HIV) transmission from MSMW to heterosexual women [37]. Thus, to model MSM accurately, we must divide MSM into men who have sex with men exclusively (MSME) and men who have sex with men and women (MSMW) because the effects of these two behaviours on public health are very different.

In this study, we simultaneously considered (1) the network heterogeneity of human sexual contacts, (2) mother-to-child (vertical) transmission and (3) MSM (i.e., MSMW and MSME) to formulate type-reproduction numbers; the type-reproduction number is defined as an extension of the basic reproduction number, ${R}_{0}$. The type-reproduction number rather than ${R}_{0}$ is required when the population is classified into several subpopulations according to epidemiological characteristics [38, 39]. Here, the type-reproduction number for type $i$, ${T}_{i}$, is the average number of secondary cases of type $i$ produced by a primary case of type $i$. Since ${T}_{i}<1\Leftrightarrow {R}_{0}<1$ regardless of type $i$, a ${T}_{i}$ value less than one indicates that STIs will be eliminated. The spread of epidemics is prevented if we effectively vaccinate at least a fraction $(1-1/{T}_{i})$ of the susceptible target type [38]. We considered four subpopulations, women, MSMW, MSME, and MSW, and derived a formula to identify which types should be targeted for public health interventions. When it is possible to concentrate vaccination on a subpopulation, the target of vaccination is not necessarily determined by the relative sizes of the type-reproduction numbers, because the cost of vaccines depends on the size of the subpopulation. However, since public health authorities do not know who is in which subpopulation, all they can do is to promote the vaccine and educate people about safe sex. It is difficult to focus promotion solely on MSME or MSMW, because they cannot be identified by the others. Therefore, even if the size of the subpopulation is small, the cost may not be so small. Thus, the type-reproduction number can be a good indicator of public health.

## Material and methods

**Fig. 1:**
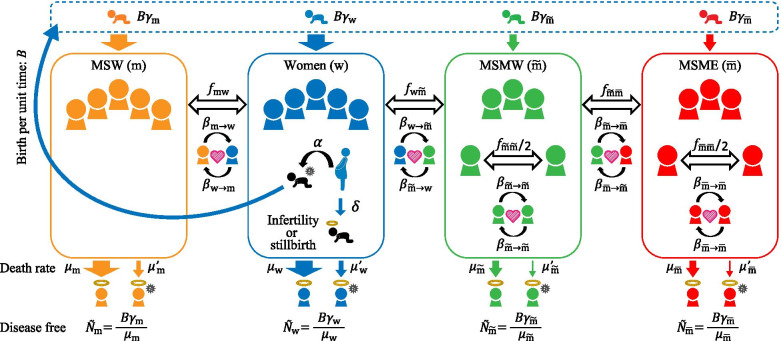
Conceptual diagram of the model. The population was divided into four subpopulations: women (w), MSMW ($\overset{\sim }{\mathrm{m}}$), MSME ($\overline{\mathrm{m} }$) and MSW (m). The subpopulation *i* individuals are born per unit time in $B{\gamma }_{i}$ and die at a rate of ${\mu }_{i}$, where ${\gamma }_{i}$ represents the proportion of subpopulation $i$ at the time of birth (${\gamma }_{\mathrm{w}}+{\gamma }_{\overset{\sim }{\mathrm{m}}}+{\gamma }_{\overline{\mathrm{m}} }+{\gamma }_{\mathrm{m}}=1$). δ is the rate of infertility or stillbirth. The number per unit time of sexual contacts between individuals in subpopulation *i* and individuals in subpopulation *j* is assumed to be ${f}_{ij}$ (white arrows in both directions). Sexual transmission occurs at a rate of ${\beta }_{i\to j}$, and mother-to-child transmission occurs at birth at a rate of $\alpha$ (black arrows)

The outline of the model is illustrated in Fig. 1. To clarify the effect of MSM, we adapt a susceptibility-infection-susceptibility (SIS) model where the population is divided into four subpopulations: $\{\mathrm{w},\overset{\sim }{\mathrm{m}},\overline{\mathrm{m} },\mathrm{m}\}$: women, MSMW, MSME, and MSW, respectively. It is assumed that a type $\overset{\sim }{\mathrm{m}}$ person has contact not only with types $\overset{\sim }{\mathrm{m}}$ and $\overline{\mathrm{m} }$ but also with type $\mathrm{w}$, while a type $\overline{\mathrm{m} }$ person has sexual contact with only types $\overset{\sim }{\mathrm{m}}$ and $\overline{\mathrm{m} }$. Here, we ignore sexual contact between women; thus, a person of type $\mathrm{w}$ can have sexual contact with persons of only types $\mathrm{m}$ and $\overset{\sim }{\mathrm{m}}$. Furthermore, the effect of generational change is also taken into consideration. Here, $B$ is the number of births per unit time, $\delta$ is the rate of infertility or stillbirths, and a newborn individual is infected at a probability of vertical transmission rate α times the female infection rate. The proportion of types of births is ${\gamma }_{\mathrm{w}}:{\gamma }_{\mathrm{m}}:{\gamma }_{\overset{\sim }{\mathrm{m}}}:{\gamma }_{\overline{\mathrm{m}} }$. Since it is not known when and how sexual orientation is determined, this model assumes that the types are fixed at birth for simplicity. The natural death rate is ${\mu }_{i}$, and the death rate for infected persons is ${\mu }_{i}^{^{\prime}}$ for type $i\in \{\mathrm{w},\overset{\sim }{\mathrm{m}},\overline{\mathrm{m} },\mathrm{m}\}$. Thus, in the equilibrium state in the absence of disease (in the case of ${I}_{i}\left(t\right)=0$, as we will see later), the population in each type is

2 $${\tilde{N }}_{i}=\frac{B{\gamma }_{i}}{{\mu }_{i}}.$$

The total number per unit time of sexual contacts between individuals of type $i$ and individuals of type $j$ is ${f}_{ij}$ for $i\ne j$ and $\frac{1}{2}{f}_{ii}$ when $i=j$. Although not specified for simplicity, ${f}_{ij}$ is a function of ${N}_{i}$ and ${N}_{j}$. From the definition, ${f}_{ij}={f}_{ji}$. From the assumptions of the model, ${f}_{\mathrm{ww}}={f}_{\mathrm{mm}}={f}_{\mathrm{m}\overset{\sim }{\mathrm{m}}}={f}_{\overset{\sim }{\mathrm{m}}\mathrm{m}}={f}_{\mathrm{w}\overline{\mathrm{m}} }={f}_{\overline{\mathrm{m}}\mathrm{w} }={f}_{\mathrm{m}\overline{\mathrm{m}} }={f}_{\overline{\mathrm{m}}\mathrm{m} }=0.$

We assume that each individual in type $i$ has a sexual activity value of ${a}_{i}$, and the value of ${a}_{i}$ follows the distribution of ${p}_{i}({a}_{i})$, where the mean of sexual activity is set to one:

3 $${\int }_{0}^{\infty }{a}_{i}{p}_{i}\left({a}_{i}\right)d{a}_{i}=1.$$

The number per unit time of sexual contacts of an individual is proportional to the individual’s sexual activity value ${a}_{i}$ (thus ${a}_{i}$ is dimensionless). It is thought that the amount of ${a}_{i}$ changes with age, but in the model, where age is ignored, it is assumed to be constant for each individual. Thus, this model assumes that the values of ${a}_{i}$ is fixed at birth. The fluctuation in sexual activity defined as Eq. (1) is given as follows:

4 $${C}_{i}= {\int }_{0}^{\infty }{a}_{i}^{2}{p}_{i}\left({a}_{i}\right)\mathrm{d}{a}_{i}.$$

It is assumed that sexual contact is well distributed, ignoring monogamy and marriage, where the rate at which individuals with ${a}_{i}$ in subpopulation $i$ sexually contact someone in subpopulation $j$ is ${a}_{i}{f}_{ij}/{N}_{i}$.

The infection dynamics are as follows: ${S}_{i}(t)$ and ${I}_{i}(t)$ represent the numbers of susceptible and infected individuals in subpopulation $i$, respectively. Assuming a susceptible-infected-susceptible model (SIS model) without carriers of immunity, the number of individuals in subpopulation $i$ is given as ${N}_{i}\left(t\right)={S}_{i}\left(t\right)+{I}_{i}(t)$. The numbers of susceptible and infected individuals in subpopulation $i$, whose sexual activities comprise infinitesimal interval $[{a}_{i},{a}_{i}+d{a}_{i}]$, are denoted as ${S}_{i}(t,{a}_{i})d{a}_{i}$ and ${I}_{i}(t,{a}_{i})d{a}_{i}$, respectively. Thus, the numbers of susceptible and infected individuals in type $i\in \{\mathrm{w},\overset{\sim }{\mathrm{m}},\overline{\mathrm{m} },\mathrm{m}\}$ are given in integral form as follows:

5 $${S}_{i}\left(t\right)={\int }_{0}^{\infty }{S}_{i}\left(t,{a}_{i}\right)d{a}_{i},$$

6 $${I}_{i}\left(t\right)={\int }_{0}^{\infty }{I}_{i}\left(t,{a}_{i}\right)d{a}_{i}.$$

The dynamics of ${S}_{i}(t,{a}_{i})$ and ${I}_{i}(t,{a}_{i})$ for $i\in \{\mathrm{w},\overset{\sim }{\mathrm{m}},\overline{\mathrm{m} },\mathrm{m}\}$ are as follows:

7 $$\frac{\partial }{\partial t}{S}_{i}\left(t,{a}_{i}\right)=B{\gamma }_{i}\frac{{S}_{\mathrm{w}}\left(t\right)+\left(1-\alpha \right)\left(1-\delta \right){I}_{\mathrm{w}}\left(t\right)}{{N}_{\mathrm{w}}\left(t\right)}{p}_{i}\left({a}_{i}\right)-{\mu }_{i}{S}_{i}\left(t,{a}_{i}\right)+{\eta }_{i}{I}_{i}\left(t,{a}_{i}\right)-\frac{{a}_{i}}{{N}_{i}\left(t\right)}{S}_{i}\left(t,{a}_{i}\right)\sum_{j\in \{\mathrm{w},\mathrm{m},\overset{\sim }{\mathrm{m}},\overline{\mathrm{m}}\}}{\beta }_{j\to i}{f}_{ij}\frac{{\Theta }_{j}\left(t\right)}{{N}_{j}(t)},$$

8 $$\frac{\partial }{\partial t}{I}_{i}\left(t,{a}_{i}\right)=B{\gamma }_{i}\frac{\alpha \left(1-\delta \right){I}_{\mathrm{w}}\left(t\right)}{{N}_{\mathrm{w}}\left(t\right)}{p}_{i}\left({a}_{i}\right)-{\mu }_{i}^{^{\prime}}{I}_{i}\left(t,{a}_{i}\right)-{\eta }_{i}{I}_{i}\left(t,{a}_{i}\right)+\frac{{a}_{i}}{{N}_{i}\left(t\right)}{S}_{i}\left(t,{a}_{i}\right)\sum_{j\in \left\{\mathrm{w},\mathrm{m},\overset{\sim }{\mathrm{m}},\overline{\mathrm{m}}\right\}}{\beta }_{j\to i}{f}_{ij}\frac{{\Theta }_{j}\left(t\right)}{{N}_{j}\left(t\right)},$$

where the parameter ${\beta }_{j\to i}$ is the probability of transmission per sexual contact from a person in subpopulation $j$ to a person subpopulation $i$, the parameter ${\eta }_{i}$ is the cure rate, and ${\Theta }_{i}\left(t\right)/{N}_{i}\left(t\right)$ represents the probability that the sexual partners are infected:

9 $${\Theta }_{i}\left(t\right)={\int }_{0}^{\infty }{I}_{i}\left(t,{a}_{i}\right){a}_{i}d{a}_{i}.$$

## Results

In the absence of infection (${I}_{i}\left(t,{a}_{i}\right)=0$), we obtain a stationary solutions of Eq. (7):

10 $${S}_{i}\left({a}_{i}\right)=\frac{B{\gamma }_{i}}{{\mu }_{i}}{p}_{i}\left({a}_{i}\right)={\tilde{N }}_{i}{p}_{i}\left({a}_{i}\right).$$

To calculate the type-reproduction number, we consider the linear dynamics of the infected state, following the methods of the previous studies (for example [39, 40]). In other words, we linearize Eq. (8) near the disease-free solution given by Eq. (10). Here, we use Eqs. (6) and (9) to derive the dynamics of ${I}_{i}(t)$ and ${\Theta }_{i}(t)$ for $i\in \{\mathrm{w},\overset{\sim }{\mathrm{m}},\overline{\mathrm{m} },\mathrm{m}\}$:

11 $$\begin{array}{c}\frac{\partial }{\partial t}{I}_i(t)=B{\gamma}_i{\alpha} \left(1-\delta \right) \frac{I_{\mathrm{w}}(t)}{{\overset{\sim}{N}}_{\mathrm{w}}}- \left({\mu}_i^{\prime} + {\eta}_{i}\right){I}_i(t)+\sum\limits_{j\in \left\{\mathrm{w},\mathrm{m},\overset{\sim}{\mathrm{m}},\overline{\mathrm{m}}\right\}} {\beta}_{j\to i}\tilde{f}_{ij} \frac{\varTheta_j(t)}{{\overset{\sim }{N}}_j},\\\frac{\partial }{\partial t}{\varTheta}_{i}(t)=B{\gamma}_{i}\alpha \left(1-\delta \right)\frac{I_{\mathrm{w}}(t)}{{\overset{\sim }{N}}_{\mathrm{w}}}-\left({\mu}_{i}^{\prime} + {\eta}_i\right){\varTheta}_i(t)+{C}_i\sum\limits_{j\in \left\{\mathrm{w},\mathrm{m},\overset{\sim }{\mathrm{m}},\overline{\mathrm{m}}\right\}}{\beta}_{j\to i}\ \tilde{f}_{ij}\frac{\varTheta_j(t)}{{\overset{\sim }{N}}_j}.\end{array}$$

Here, ${\tilde{f }}_{ij}={f}_{ij}\left({\tilde{N }}_{i},{\tilde{N }}_{j}\right)$. Thus, the situation with very few infected persons is represented by a linear differential equation system closed by eight variables. According to the traditional method of calculating the type-reproduction number by Diekmann et al. [39], the Jacobi matrix $J$ presented in Eq. (11) is divided into part $T$, which is related to infection of the target type, and part $Q=J-T$, and the dominant eigenvalue of $-{TQ}^{-1}$ is calculated. Thus, we need to perform complicated algebraic operations on an eight-dimensional matrix.

**Fig. 2:**
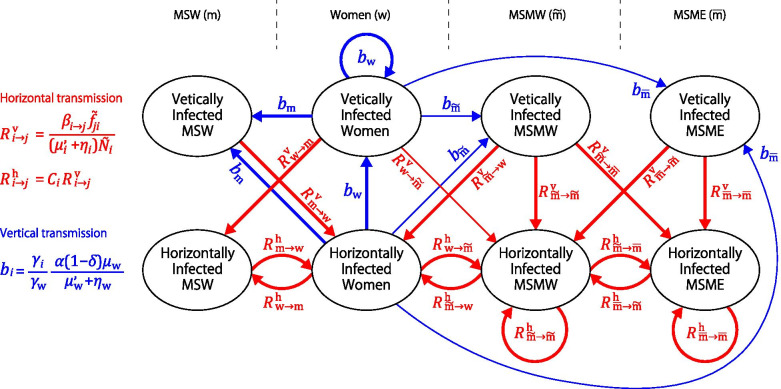
Network diagram among eight types of infection states. The red arrows represent the type-reproduction numbers of all sexual (horizontal) transmission events that occur between subpopulations. The blue arrows indicate the type-reproduction numbers of all mother-to-child (vertical) transmission events caused by infected women. The thin arrows indicate that the contribution is small when the proportion of homosexual individuals is small (see Table 3)

We use the network diagram method according to the work by Lewis et al. [41], which is an intuitive and easy-to-understand method. Infected persons of each type are further divided into those by mother-to-child (vertical) transmission and those by sexual (horizontal) transmission. Thus, there are eight infection states, and we consider their network (see Fig. 2). In Fig. 2, red arrows indicate that infected persons in the state at the end of the arrow are infected from infected persons in the state at the beginning of the arrow. Blue arrows indicate that infected persons in the state at the ends of the arrow are born from an infected mother in the state at the beginning of the arrow. The quantities near the arrows give the number of new infections born per unit time divided by the duration of the original infected state. ${R}_{ij}^{\mathrm{v}}$ and ${R}_{ij}^{\mathrm{h}}$ represent the average number of the original persons in subpopulation $j$ infected through sexual transmissions from a typical *vertically* and *horizontally* infected person in subpopulation $i$, respectively, as follows [32]:

12 $${R}_{i\to j}^{\mathrm{v}}=\frac{{\beta }_{i\to \mathrm{j}}{f}_{ji}}{\left({\mu }_{i}^{^{\prime}}+{\eta }_{i}\right){\tilde{N }}_{i}}, {R}_{i\to j}^{\mathrm{h}}={C}_{i}{R}_{i\to j}^{\mathrm{v}},$$

where ${C}_{i}$ is the fluctuation of sexual activity ${a}_{i}$ in subpopulation $i$ (see Eq. (4)), and ${b}_{i}$ represents *direct* vertical transmission as follows:

13 $${b}_{i}=\frac{{\gamma }_{i}}{{\gamma }_{\mathrm{w}}}\frac{\alpha \left(1-\delta \right){\mu }_{\mathrm{w}}}{{\mu }_{\mathrm{w}}^{^{\prime}}+{\eta }_{\mathrm{w}}}.$$

The network diagram in Fig. 2 is represented by the following transition matrix:

14 $$A=\left(\begin{array}{cccccccc}{b}_{\mathrm{w}}& {b}_{\mathrm{w}}& 0& 0& 0& 0& 0& 0\\ 0& 0& {R}_{\overset{\sim }{\mathrm{m}}\to \mathrm{w}}^{\mathrm{v}}& {R}_{\overset{\sim }{\mathrm{m}}\to \mathrm{w}}^{\mathrm{h}}& 0& 0& {R}_{\mathrm{m}\to \mathrm{w}}^{\mathrm{v}}& {R}_{\mathrm{m}\to \mathrm{w}}^{\mathrm{h}}\\ {b}_{\overset{\sim }{\mathrm{m}}}& {b}_{\overset{\sim }{\mathrm{m}}}& 0& 0& 0& 0& 0& 0\\ {R}_{\mathrm{w}\to \overset{\sim }{\mathrm{m}}}^{\mathrm{v}}& {R}_{\mathrm{w}\to \overset{\sim }{\mathrm{m}}}^{\mathrm{h}}& {R}_{\overset{\sim }{\mathrm{m}}\to \overset{\sim }{\mathrm{m}}}^{\mathrm{v}}& {R}_{\overset{\sim }{\mathrm{m}}\to \overset{\sim }{\mathrm{m}}}^{\mathrm{h}}& {R}_{\overline{\mathrm{m}}\to \overset{\sim }{\mathrm{m}} }^{\mathrm{v}}& {R}_{\overline{\mathrm{m}}\to \overset{\sim }{\mathrm{m}} }^{\mathrm{h}}& 0& 0\\ {b}_{\overline{\mathrm{m}} }& {b}_{\overline{\mathrm{m}} }& 0& 0& 0& 0& 0& 0\\ 0& 0& {R}_{\overset{\sim }{\mathrm{m}}\to \overline{\mathrm{m}} }^{\mathrm{v}}& {R}_{\overset{\sim }{\mathrm{m}}\to \overline{\mathrm{m}} }^{\mathrm{h}}& {R}_{\overline{\mathrm{m} }\to \overline{\mathrm{m}} }^{\mathrm{v}}& {R}_{\overline{\mathrm{m} }\to \overline{\mathrm{m}} }^{\mathrm{h}}& 0& 0\\ {b}_{\mathrm{m}}& {b}_{\mathrm{m}}& 0& 0& 0& 0& 0& 0\\ {R}_{\mathrm{w}\to \mathrm{m}}^{\mathrm{v}}& {R}_{\mathrm{w}\to \mathrm{m}}^{\mathrm{h}}& 0& 0& 0& 0& 0& 0\end{array}\right).$$

The dominant eigenvalue of the matrix $A$ gives the basic reproduction number, but it cannot be given in an analytical form because the characteristic equation becomes a quartic equation. On the other hand, the type-reproduction number can be written in a relatively simple formula. For example, if sexually infected females are the focus, the target matrix $B$ has only nonzero entries ${B}_{2k}={A}_{2k}$ for $1\le \mathrm{k}\le 8$. In this case, the type-reproduction number is given by the dominant eigenvalue of the matrix $B(I-A+B)$ [39]. Since the rank of the matrix $B(I-A+B)$ is one, the calculation of eigenvalues is easy. The type-reproduction number can be calculated using numerical-analysis software (the Mathematica source code is included in the Additional file 1). By performing some troublesome formula transformations, we obtain the type-reproduction numbers of persons in the four subpopulations who are infected sexually as follows if the denominators are positive:

15 $$\begin{array}{l}{T}_{\mathrm{w}}={A}_{\mathrm{m}}{R}_{\mathrm{m}\to \mathrm{w}}^{\mathrm{v}}+{A}_{\overset{\sim }{\mathrm{m}}}{R}_{\overset{\sim }{\mathrm{m}}\to \mathrm{w}}^{\mathrm{v}}+{\left({R}_{\mathrm{w}\to \mathrm{m}}^{\mathrm{h}}+{A}_{\mathrm{w}}{R}_{\mathrm{w}\to \mathrm{m}}^{\mathrm{v}}\right)R}_{\mathrm{m}\to \mathrm{w}}^{\mathrm{h}}+\frac{{R}_{\mathrm{w}\to \overset{\sim }{\mathrm{m}}}^{\mathrm{h}}+{A}_{\mathrm{w}}{R}_{\mathrm{w}\to \overset{\sim }{\mathrm{m}}}^{\mathrm{v}}+{A}_{\overset{\sim }{\mathrm{m}}}{R}_{\overset{\sim }{\mathrm{m}}\to \overset{\sim }{\mathrm{m}}}^{\mathrm{v}}+{A}_{\overline{\mathrm{m}}}{R }_{\overline{\mathrm{m}}\to \overset{\sim }{\mathrm{m}} }^{\mathrm{v}}+\frac{{A}_{\overset{\sim }{\mathrm{m}}}{R}_{\overset{\sim }{\mathrm{m}}\to \overline{\mathrm{m}} }^{\mathrm{v}}+{A}_{\overline{\mathrm{m}}}{R }_{\overline{\mathrm{m} }\to \overline{\mathrm{m}} }^{\mathrm{v}}}{1-{R}_{\overline{\mathrm{m} }\to \overline{\mathrm{m}} }^{\mathrm{h}}}{R}_{\overline{\mathrm{m}}\to \overset{\sim }{\mathrm{m}} }^{\mathrm{h}}}{1-{R}_{\overset{\sim }{\mathrm{m}}\to \overset{\sim }{\mathrm{m}}}^{\mathrm{h}}-\frac{{R}_{\overset{\sim }{\mathrm{m}}\to \overline{\mathrm{m}} }^{\mathrm{h}}{R}_{\overline{\mathrm{m}}\to \overset{\sim }{\mathrm{m}} }^{\mathrm{h}}}{1-{R}_{\overline{\mathrm{m} }\to \overline{\mathrm{m}} }^{\mathrm{h}}}}{R}_{\overset{\sim }{\mathrm{m}}\to \mathrm{w}}^{\mathrm{h}},\\ {T}_{\overset{\sim }{\mathrm{m}}}={R}_{\overset{\sim }{\mathrm{m}}\to \overset{\sim }{\mathrm{m}}}^{\mathrm{h}}+\frac{{R}_{\overset{\sim }{\mathrm{m}}\to \overline{\mathrm{m}} }^{\mathrm{h}}{R}_{\overline{\mathrm{m}}\to \overset{\sim }{\mathrm{m}} }^{\mathrm{h}}}{1-{R}_{\overline{\mathrm{m} }\to \overline{\mathrm{m}} }^{\mathrm{h}}}+\frac{{R}_{\overset{\sim }{\mathrm{m}}\to \mathrm{w}}^{\mathrm{h}}\left({R}_{\mathrm{w}\to \overset{\sim }{\mathrm{m}}}^{\mathrm{h}}+{A}_{\mathrm{w}}{R}_{\mathrm{w}\to \overset{\sim }{\mathrm{m}}}^{\mathrm{v}}+{A}_{\overset{\sim }{\mathrm{m}}}{R}_{\overset{\sim }{\mathrm{m}}\to \overset{\sim }{\mathrm{m}}}^{\mathrm{v}}+{A}_{\overline{\mathrm{m}}}{R }_{\overline{\mathrm{m}}\to \overset{\sim }{\mathrm{m}} }^{\mathrm{v}}+\left({A}_{\overset{\sim }{\mathrm{m}}}{R}_{\overset{\sim }{\mathrm{m}}\to \overline{\mathrm{m}} }^{\mathrm{v}}+{A}_{\overline{\mathrm{m}}}{R }_{\overline{\mathrm{m} }\to \overline{\mathrm{m}} }^{\mathrm{v}}\right){R}_{\overline{\mathrm{m}}\to \overset{\sim }{\mathrm{m}} }^{\mathrm{h}}/\left(1-{R}_{\overline{\mathrm{m} }\to \overline{\mathrm{m}} }^{\mathrm{h}}\right)\right)}{1-{A}_{\overset{\sim }{\mathrm{m}}}{R}_{\overset{\sim }{\mathrm{m}}\to \mathrm{w}}^{\mathrm{v}}-{A}_{\mathrm{m}}{R}_{\mathrm{m}\to \mathrm{w}}^{\mathrm{v}}-\left({R}_{\mathrm{w}\to \mathrm{m}}^{\mathrm{h}}+{A}_{\mathrm{w}}{R}_{\mathrm{w}\to \mathrm{m}}^{\mathrm{v}}\right){R}_{\mathrm{m}\to \mathrm{w}}^{\mathrm{h}}},\\ \begin{array}{c}{T}_{\overline{m} }={R}_{\overline{m }\to \overline{m} }^{h}+\frac{{R}_{\overline{\mathrm{m}}\to \overset{\sim }{\mathrm{m}} }^{\mathrm{h}}\left[{R}_{\overset{\sim }{\mathrm{m}}\to \overline{\mathrm{m}} }^{\mathrm{h}}+\frac{{R}_{\overset{\sim }{\mathrm{m}}\to \mathrm{w}}^{\mathrm{h}}\left({A}_{\overset{\sim }{\mathrm{m}}}{R}_{\overset{\sim }{\mathrm{m}}\to \overline{\mathrm{m}} }^{\mathrm{v}}+{A}_{\overline{\mathrm{m}}}{R }_{\overline{\mathrm{m} }\to \overline{\mathrm{m}} }^{\mathrm{v}}\right)}{1-{A}_{\overset{\sim }{\mathrm{m}}}{R}_{\overset{\sim }{\mathrm{m}}\to \mathrm{w}}^{\mathrm{v}}-{A}_{\mathrm{m}}{R}_{\mathrm{m}\to \mathrm{w}}^{\mathrm{v}}-({R}_{\mathrm{w}\to \mathrm{m}}^{\mathrm{h}}+{A}_{\mathrm{w}}{R}_{\mathrm{w}\to \mathrm{m}}^{\mathrm{v}}){R}_{\mathrm{m}\to \mathrm{w}}^{\mathrm{h}}} \right]}{1-{R}_{\overset{\sim }{\mathrm{m}}\to \overset{\sim }{\mathrm{m}}}^{\mathrm{h}}-{R}_{\overset{\sim }{\mathrm{m}}\to \mathrm{w}}^{\mathrm{h}}\left({R}_{\mathrm{w}\to \overset{\sim }{\mathrm{m}}}^{\mathrm{h}}+{A}_{\mathrm{w}}{R}_{\mathrm{w}\to \overset{\sim }{\mathrm{m}}}^{\mathrm{v}}+{A}_{\overset{\sim }{\mathrm{m}}}{R}_{\overset{\sim }{\mathrm{m}}\to \overset{\sim }{\mathrm{m}}}^{\mathrm{v}}+{A}_{\overline{\mathrm{m}}}{R }_{\overline{\mathrm{m}}\to \overset{\sim }{\mathrm{m}} }^{\mathrm{v}}\right)},\\ {T}_{\mathrm{m}}=\frac{{R}_{\mathrm{m}\to \mathrm{w}}^{\mathrm{h}}({R}_{\mathrm{w}\to \mathrm{m}}^{\mathrm{h}}+{A}_{\mathrm{w}}{R}_{\mathrm{w}\to \mathrm{m}}^{\mathrm{v}})/(1-{A}_{\overset{\sim }{\mathrm{m}}}{R}_{\overset{\sim }{\mathrm{m}}\to \mathrm{w}}^{\mathrm{v}}-{A}_{\mathrm{m}}{R}_{\mathrm{m}\to \mathrm{w}}^{\mathrm{v}})}{1-\frac{{R}_{\mathrm{w}\to \overset{\sim }{\mathrm{m}}}^{\mathrm{h}}+{A}_{\mathrm{w}}{R}_{\mathrm{w}\to \overset{\sim }{\mathrm{m}}}^{\mathrm{v}}+{A}_{\overset{\sim }{\mathrm{m}}}{R}_{\overset{\sim }{\mathrm{m}}\to \overset{\sim }{\mathrm{m}}}^{\mathrm{v}}+{A}_{\overline{\mathrm{m}}}{R }_{\overline{\mathrm{m}}\to \overset{\sim }{\mathrm{m}} }^{\mathrm{v}}+({A}_{\overset{\sim }{\mathrm{m}}}{R}_{\overset{\sim }{\mathrm{m}}\to \overline{\mathrm{m}} }^{\mathrm{v}}+{A}_{\overline{\mathrm{m}}}{R }_{\overline{\mathrm{m} }\to \overline{\mathrm{m}} }^{\mathrm{v}}){R}_{\overline{\mathrm{m}}\to \overset{\sim }{\mathrm{m}} }^{\mathrm{h}}/\left(1-{R}_{\overline{\mathrm{m} }\to \overline{\mathrm{m}} }^{\mathrm{h}}\right)}{\left(1-{R}_{\overset{\sim }{\mathrm{m}}\to \overset{\sim }{\mathrm{m}}}^{\mathrm{h}}-\frac{{R}_{\overset{\sim }{\mathrm{m}}\to \overline{\mathrm{m}} }^{\mathrm{h}}{R}_{\overline{\mathrm{m}}\to \overset{\sim }{\mathrm{m}} }^{\mathrm{h}}}{1-{R}_{\overline{\mathrm{m} }\to \overline{\mathrm{m}} }^{\mathrm{h}}}\right)\left(1-{A}_{\overset{\sim }{\mathrm{m}}}{R}_{\overset{\sim }{\mathrm{m}}\to \mathrm{w}}^{\mathrm{v}}-{A}_{\mathrm{m}}{R}_{\mathrm{m}\to \mathrm{w}}^{\mathrm{v}}\right)}}.\end{array}\end{array}$$

Here, ${A}_{i}$ represents the average number of persons in each subpopulation ($i\in\left\{\mathrm{w},\mathrm{m},\overset{\sim }{\mathrm{m}},\overline{\mathrm{m}}\right\}$) who are infected through *consecutive* vertical transmissions from a sexually infected adult woman:

$${A}_{i}=\frac{{b}_{i}}{1-{b}_{\mathrm{w}}}.$$

Note that in Eq. (15), ${R}_{i\to j}^{v}$ are always multiplied by ${A}_{i}$, such as ${A}_{i}{R}_{i\to j}^{v}$. Therefore, no vertical transmission ($\alpha =0$) always makes ${A}_{i}{R}_{i\to j}^{v}=0$, even if ${R}_{i\to j}^{v}$ is positive. If the denominators in Eq. (15) are not positive, they are not well defined, which means that infectious diseases are not extinct although the infection rate for the type of interest can approach zero. In particular, if the STI increases in only MSM,

16 $${R}_{\overset{\sim }{\mathrm{m}}\to \overset{\sim }{\mathrm{m}}}^{\mathrm{h}}+\frac{{R}_{\overset{\sim }{\mathrm{m}}\to \overline{\mathrm{m}} }^{\mathrm{h}}{R}_{\overline{\mathrm{m}}\to \overset{\sim }{\mathrm{m}} }^{\mathrm{h}}}{1-{R}_{\overline{\mathrm{m} }\to \overline{\mathrm{m}} }^{\mathrm{h}}}\ge 1,$$

the infection cannot be controlled without measures for MSM. On the other hand, if Eq. (16) does not hold, it is difficult to eradicate infections by targeting only MSM.

**Fig. 3:**
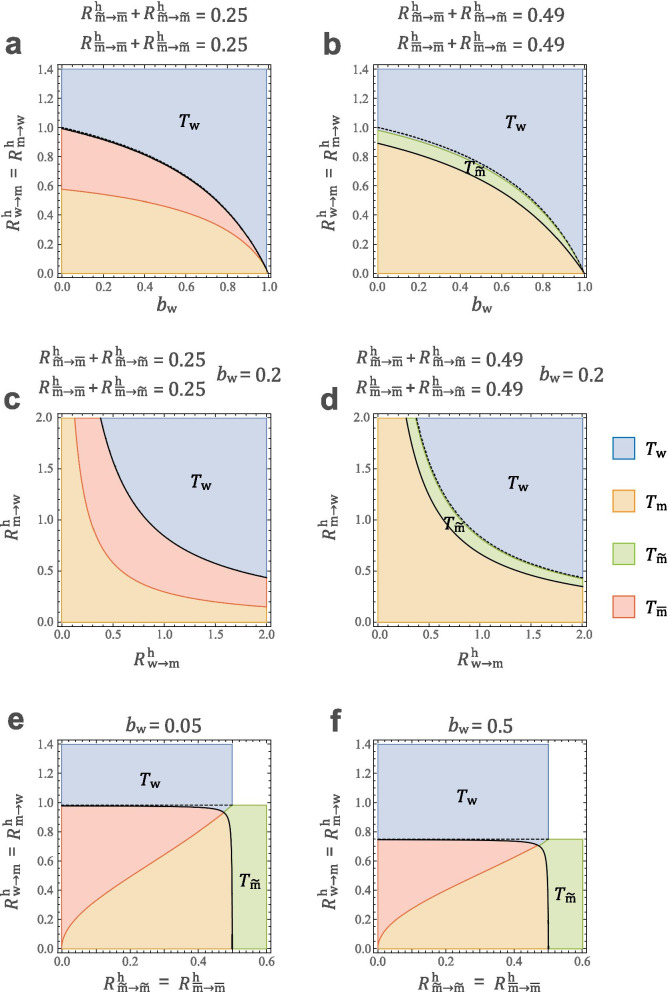
The phase plane of the STI survival region and smallest type-reproduction numbers. The region above the black bold curve indicates the survival region of STIs (i.e., ${T}_{\mathrm{w}},{T}_{\mathrm{m}},{T}_{\overset{\sim }{\mathrm{m}}},{T}_{\overline{\mathrm{m}} }>1$), and the four colours indicate which type-reproduction numbers are the smallest. The black dashed curves indicate that ${T}_{\overset{\sim }{\mathrm{m}}}$ is not well defined above them. In the cases of a and c the dashed curves are identical to the bold ones. In the colourless area, no type-reproduction numbers are well defined. The other parameters are set as $({\gamma }_{\mathrm{w}},{\gamma }_{\mathrm{m}},{\gamma }_{\overset{\sim }{\mathrm{m}}},{\gamma }_{\overline{\mathrm{m}} })=(\mathrm{0.5,0.48,0},\mathrm{01,0.01})$

Figure 3 illustrates some typical results, and we make additional assumptions as follows:

- The total numbers per unit time of sexual contacts are given as
  17 $$\begin{array}{c}{f}_{\mathrm{wm}}={f}_{\mathrm{mw}}={k}_{\mathrm{hetero}}{N}_{\mathrm{m}}, {f}_{\mathrm{w}\overset{\sim }{\mathrm{m}}}={f}_{\overset{\sim }{\mathrm{m}}\mathrm{w}}={k}_{\mathrm{hetero}}{N}_{\overset{\sim }{\mathrm{m}}},\\ {f}_{\overset{\sim }{\mathrm{m}}\overset{\sim }{\mathrm{m}}}={k}_{\mathrm{homo}}\frac{{N}_{\overset{\sim }{\mathrm{m}}}^{2}}{{N}_{\overset{\sim }{\mathrm{m}}}+{N}_{\overline{\mathrm{m}}} }, {f}_{\overline{\mathrm{m} }\overline{\mathrm{m}} }={k}_{\mathrm{homo}}\frac{{N}_{\overline{\mathrm{m}} }^{2}}{{N}_{\overset{\sim }{\mathrm{m}}}+{N}_{\overline{\mathrm{m}}} },\\ {f}_{\overset{\sim }{\mathrm{m}}\overline{\mathrm{m}} }={f}_{\overline{\mathrm{m}}\overset{\sim }{\mathrm{m}} }={k}_{\mathrm{homo}}\frac{{N}_{\overset{\sim }{\mathrm{m}}}{N}_{\overline{\mathrm{m}}}}{{N }_{\overset{\sim }{\mathrm{m}}}+{N}_{\overline{\mathrm{m}}} }.\end{array}$$
- As a result, for a man of types $\mathrm{m}$ and $\overset{\sim }{\mathrm{m}}$ and women, the average number of heterosexual contacts per unit time is ${k}_{\mathrm{hetero}}$ and ${k}_{\mathrm{hetero}}{N}_{\mathrm{m}}/{N}_{\mathrm{w}}$, respectively. They are approximately equal to each other because the sex ratio is approximately equal. For a man of types $\overset{\sim }{\mathrm{m}}$ and $\overline{\mathrm{m} }$, the average number of homosexual contacts per unit time is ${k}_{\mathrm{homo}}$.
- Life history parameters (${\gamma }_{i},{\eta }_{i},{\mu }_{i}^{^{\prime}}$) and the fluctuation in sexual activity are set to ${C}_{i}=3$ for all categories $i\in \left\{\mathrm{w},\mathrm{m},\overset{\sim }{\mathrm{m}},\overline{\mathrm{m}}\right\}$. The observational basis for the value of C will be explained later. Increasing ${C}_{i}$ reduces the effect of mother-to-child transmission (${b}_{\mathrm{w}}$), but the results do not change qualitatively.
- Consider only three values of infection rates: female to male (${\beta }_{\mathrm{w}\to \mathrm{m}}={\beta }_{\mathrm{w}\to \overset{\sim }{\mathrm{m}}}={\beta }_{\mathrm{w}\to \overline{\mathrm{m}} }$), male to female (${\beta }_{\mathrm{m}\to \mathrm{w}}={\beta }_{\overset{\sim }{\mathrm{m}}\to \mathrm{w}}={\beta }_{\overline{\mathrm{m}}\to \mathrm{w} }$), and male to male (${\beta }_{\overset{\sim }{\mathrm{m}}\to \overset{\sim }{\mathrm{m}}}={\beta }_{\overline{\mathrm{m}}\to \overset{\sim }{\mathrm{m}} }={\beta }_{\overset{\sim }{\mathrm{m}}\to \overline{\mathrm{m}} }={\beta }_{\overline{\mathrm{m} }\to \overline{\mathrm{m}} }$).

**Fig. 4:**
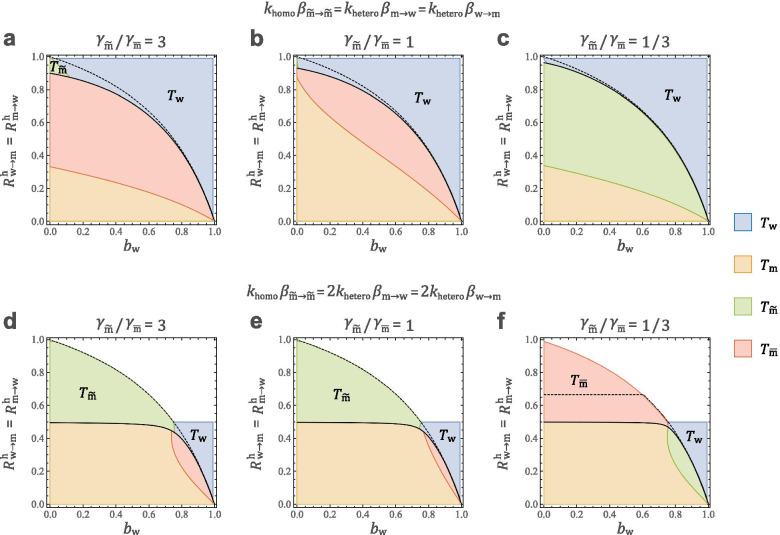
The phase plane of the STI survival region and smallest type-reproduction numbers. The region above the black bold curve indicates the survival region of STIs (i.e., ${T}_{\mathrm{w}},{T}_{\mathrm{m}},{T}_{\overset{\sim }{\mathrm{m}}},{T}_{\overline{\mathrm{m}} }>1$), and the four colours indicate which type-reproduction numbers are the smallest. The black dashed curves indicate that ${T}_{\overset{\sim }{\mathrm{m}}}$ is not well defined above them. In the colourless area, no type-reproduction numbers are well defined. In the top three figures, homosexual and heterosexual transmission rates are the same, whereas in the bottom three figures, homosexual transmission is twice as high as heterosexual transmission. The ratio of MSMW and MSME is set as (a,d) ${\gamma }_{\overset{\sim }{\mathrm{m}}}/{\gamma }_{\overline{\mathrm{m}} }=3$, (b,e) ${\gamma }_{\overset{\sim }{\mathrm{m}}}/{\gamma }_{\overline{\mathrm{m}} }=1$ and (c,f) ${\gamma }_{\overset{\sim }{\mathrm{m}}}/{\gamma }_{\overline{\mathrm{m}} }=1/3$ (set ${\gamma }_{\overset{\sim }{\mathrm{m}}}+{\gamma }_{\overline{\mathrm{m}} }=0.02$)

Under the above assumption, the relative sizes of ${R}_{i\to j}^{\mathrm{v}}$ and ${A}_{i}$ are as shown in Table 2. In the phase plane in Fig. 3, the region above the bold curve indicates the persistence region of STIs (i.e., ${T}_{\mathrm{w}},{T}_{\mathrm{m}},{T}_{\overset{\sim }{\mathrm{m}}},{T}_{\overline{\mathrm{m}} }>1$), the four colours indicate which type-reproduction numbers are the smallest, and the colourless region indicates that all ${T}_{i}$ are not well defined. The dashed curves indicate that ${T}_{\overset{\sim }{\mathrm{m}}}$ is not well defined above them (in Fig. 3a and c, the dashed curves coincide with the solid ones). If there are relatively few sexual contacts between men, it is more effective to focus infection control on women (w) (${R}_{\overset{\sim }{\mathrm{m}}\to \overline{\mathrm{m}} }^{\mathrm{h}}={R}_{\overset{\sim }{\mathrm{m}}\to \overset{\sim }{\mathrm{m}}}^{\mathrm{h}}={R}_{\overline{\mathrm{m} }\to \overline{\mathrm{m}} }^{\mathrm{h}}={R}_{\overline{\mathrm{m}}\to \overset{\sim }{\mathrm{m}} }^{\mathrm{h}}=0.25$ in Fig. 3a and c) than on men. In this case, measures focused only on MSMW will not be able to suppress the STIs, because ${T}_{\overset{\sim }{\mathrm{m}}}$ is not well defined. On the other hand, if there are relatively more sexual contacts between men, there are some parameter areas where focusing infection control on MSMW ($\overset{\sim }{\mathrm{m}}$) is most effective (${R}_{\overset{\sim }{\mathrm{m}}\to \overline{\mathrm{m}} }^{\mathrm{h}}={R}_{\overset{\sim }{\mathrm{m}}\to \overset{\sim }{\mathrm{m}}}^{\mathrm{h}}={R}_{\overline{\mathrm{m} }\to \overline{\mathrm{m}} }^{\mathrm{h}}={R}_{\overline{\mathrm{m}}\to \overset{\sim }{\mathrm{m}} }^{\mathrm{h}}=0.49$ in Fig. 3b and d). However, such a parameter range is narrow, and ${T}_{\overset{\sim }{\mathrm{m}}}$ is not well define in most of the blue regions where ${T}_{\mathrm{w}}$ is minimized. Moreover, Fig. 3e and f show that it would be most efficient to focus prevention measures on women (w) if there is little transmission among men and on MSME ($\overline{\mathrm{m} }$) otherwise. If there are many cases of both transmission types, infection cannot be suppressed by taking measures for only one subpopulation. This suggests that it is important to simultaneously prevent both homosexual and heterosexual transmission to suppress STIs. Moreover, in Fig. 4, we compare the influence of MSMW and MSME. Figure 4d-f show that when homosexuals contribute more to infectious diseases than heterosexuals, higher proportions of MSME require more measures against MSME than MSMW. On the other hand, Fig. 4a-c show that if the contributions to homosexual and heterosexual infections are equal, measures for women are important in any case.

**Table 2:** Relative sizes of the parameters used to create Figs. 3 and 4. Since the sex ratio is approximately 1:1 (${\upgamma }_{\mathrm{m}}\cong {\upgamma }_{\mathrm{w}}$) and the life history parameters are almost equal in the four sex categories, the magnitude relations are roughly established. In addition, the relative magnitude of ${R}_{i\to j}^{\mathrm{h}}$ is obtained from ${R}_{i\to j}^{\mathrm{h}}={C}_{i}{R}_{i\to j}^{\mathrm{v}}$. Since ${\upgamma }_{\overset{\sim }{\mathrm{m}}},{\upgamma }_{\overline{\mathrm{m}} }<{\upgamma }_{w},{\upgamma }_{\mathrm{m}}$, the route (${R}_{\mathrm{w}\to \overset{\sim }{\mathrm{m}}}^{\mathrm{v}}$) of sexual transmission from women to MSM can be said to be “narrower” than the opposite route (${R}_{\overset{\sim }{\mathrm{m}}\to w}^{\mathrm{v}}$). The effect (${A}_{\overset{\sim }{\mathrm{m}}},{A}_{\overline{\mathrm{m}} }$) of the vertical route from mother to MSM is also weaker than other mother-to-child transmissions (${A}_{\mathrm{w}},{A}_{\mathrm{m}}$)

	${R}_{\mathrm{w}\to \mathrm{m}}^{\mathrm{v}}$	${R}_{\mathrm{m}\to \mathrm{w}}^{\mathrm{v}}$	${R}_{\mathrm{w}\to \overset{\sim }{\mathrm{m}}}^{\mathrm{v}}$	${R}_{\overset{\sim }{\mathrm{m}}\to w}^{\mathrm{v}}$
Relative size	${\beta }_{\mathrm{w}\to \mathrm{m}}{k}_{\mathrm{hetero}}$	${\beta }_{\mathrm{m}\to \mathrm{w}}{k}_{\mathrm{hetero}}$	${\beta }_{\mathrm{w}\to \mathrm{m}}{k}_{\mathrm{hetero}}\frac{{\upgamma }_{\overset{\sim }{\mathrm{m}}}}{{\upgamma }_{\mathrm{m}}}$	${\beta }_{\mathrm{m}\to \mathrm{w}}{k}_{\mathrm{hetero}}$
	${R}_{\overset{\sim }{\mathrm{m}}\to \overline{\mathrm{m}} }^{\mathrm{v}}$	${R}_{\overline{\mathrm{m}}\to \overset{\sim }{\mathrm{m}} }^{\mathrm{v}}$	${R}_{\overset{\sim }{\mathrm{m}}\to \overset{\sim }{\mathrm{m}}}^{\mathrm{v}}$	${R}_{\overline{\mathrm{m} }\to \overline{\mathrm{m}} }^{\mathrm{v}}$
Relative size	${\beta }_{\overset{\sim }{\mathrm{m}}\overset{\sim }{\mathrm{m}}}{k}_{\mathrm{homo}}\frac{{\upgamma }_{\overline{\mathrm{m}}}}{{\upgamma }_{\overset{\sim }{\mathrm{m}}}+{\upgamma }_{\overline{\mathrm{m}}} }$	${\beta }_{\overset{\sim }{\mathrm{m}}\overset{\sim }{\mathrm{m}}}{k}_{\mathrm{homo}}\frac{{\upgamma }_{\overset{\sim }{\mathrm{m}}}}{{\upgamma }_{\overset{\sim }{\mathrm{m}}}+{\upgamma }_{\overline{\mathrm{m}}} }$	${\beta }_{\overset{\sim }{\mathrm{m}}\overset{\sim }{\mathrm{m}}}{k}_{\mathrm{homo}}\frac{{\upgamma }_{\overset{\sim }{\mathrm{m}}}}{{\upgamma }_{\overset{\sim }{\mathrm{m}}}+{\upgamma }_{\overline{\mathrm{m}}} }$	${\beta }_{\overset{\sim }{\mathrm{m}}\overset{\sim }{\mathrm{m}}}{k}_{\mathrm{homo}}\frac{{\upgamma }_{\overline{\mathrm{m}}}}{{\upgamma }_{\overset{\sim }{\mathrm{m}}}+{\upgamma }_{\overline{\mathrm{m}}} }$
	${A}_{\mathrm{w}}=\frac{{b}_{\mathrm{w}}}{1-{b}_{\mathrm{w}}}$	${A}_{\mathrm{m}}=\frac{{b}_{\mathrm{m}}}{1-{b}_{\mathrm{w}}}$	${A}_{\overset{\sim }{\mathrm{m}}}=\frac{{b}_{\overset{\sim }{\mathrm{m}}}}{1-{b}_{\mathrm{w}}}$	${A}_{\overline{\mathrm{m}} }=\frac{{b}_{\overline{\mathrm{m}}} }{1-{b}_{\mathrm{w}}}$
Relative size	$\alpha$	$\alpha$	$\frac{{\gamma }_{\overset{\sim }{\mathrm{m}}}}{{\gamma }_{\mathrm{m}}} \alpha$	$\frac{{\gamma }_{\overline{\mathrm{m}}}}{{\gamma }_{\mathrm{m}}} \alpha$

## Discussion

In our previous paper [31], we proposed a simple STI model with heterosexual and vertical transmission and studied their mutual effect on the spread of STIs. In this model, people were divided into two types by sex. Moreover, we extended this model to include a juvenile type and showed that it is not necessary to include the juvenile type in the model because its effect can be mathematically reduced to postnatal effects [32]. In this paper, we formulated a type-reproduction number for the STI model that simultaneously considered (1) the network heterogeneity of human sexual contacts, (2) mother-to-child (vertical) transmission and (3) MSMW and MSME. These three factors greatly influence the spread of STIs, and we expect that the current approach will contribute to a comprehensive understanding of STI infection dynamics. It should be emphasized that the result given by Eq. (15) does not depend on the details of the model, such as the addition of the childhood stage.

In the current model, we assume a well-mixed population without consideration of the specific network structure, and we assume that each individual has a different level of sexual activity. This type of approximation is good for epidemic models with complex networks (e.g., [31, 42]). Although our model does not take into account the details of personal relationships (e.g., marital status, distinguishing between primary and casual sexual partners, repeated sexual contacts, and parent–child relationships), the result provides a good reference theory for complicated situations. Our model can reveal various trends in the population by changing the parameters. Several types of sexual contact (oral, anal and genital) can be considered differences in the level of sexual activity. Moreover, the transmission rates (${\beta }_{i\to \mathrm{j}}$) are dependent on sexual culture, which changes over time. In this study, we neglected women who have sex with women (WSW). Although WSW can potentially transmit STIs from current and prior male and female partners [42], it is unlikely that the WSW sexual network is a large reservoir of STIs, in contrast with the MSM network, because the prevalence of STIs in women who have sex with women exclusively (WSWE) is not higher than the prevalence in heterosexual women for many STIs [43, 44]. We considered only MSM, which has been confirmed to contribute significantly to the spread of STIs.

This model assumed that MSMW and MSME are innate, and the proportion of the sum of MSMW and MSME was set to 4% according to the previous studies shown in Table 1. Again, it is difficult to distinguish between MSMW and MSME, and the population ratio of MSMW and MSME cannot be determined definitively. This problem makes the estimation of the cost-effectiveness of prevention measures for each subpopulation difficult. The fluctuation of sexual activity is set to ${C}_{i}=3$ for all subpopulations according to the data of previous studies (see Table 3) [28, 45–52]. Although the values of ${C}_{i}$ may be slightly larger, our result does not change qualitatively when ${C}_{i}$ increases. There was almost no difference in ${C}_{\mathrm{i}}$ between men and women in Finland and Russia. In the UK, there was a large difference between men and women, with ${C}_{\mathrm{m}}=68$ for men and ${C}_{\mathrm{w}}=15$ for women. The *C* in Japan is large because the surveys cover all ages; thus, the variance in the total number of sexual partners is large. However, *C* in Japan may not be that large in reality since sexual activity in the model is assumed to be innate.

**Table 3:** The fluctuation of sexual activity derived based on data in previous studies. Here, we regard the number of people having sexual contact as sexual activity

Countries and references	Period	Male			Female		
		Variance	Mean	C	Variance	Mean	C
Britain 1992 [47]	lifetime	6575	9.9	**68.1**	165	3.4	**15.3**
Britain 2000 [48]	lifetime	1239	12.7	**8.7**	94	6.5	**3.2**
Sweden 1996 [49]	lifetime	1173	14.5	**6.6**	70	6.7	**2.6**
Finland 1971 [50]	lifetime	352	11	**3.9**	16	2.6	**3.4**
Finland 1992 [50]	lifetime	369	13.8	**2.9**	67	5.2	**3.5**
Finland 1999 [50]	lifetime	420	14.4	**3.0**	88	6.6	**3.0**
Russia 1996 [50]	lifetime	310	11.5	**3.3**	31	4.2	**2.8**
Rakai [51]	last year	1.23	1.28	**1.8**	0.27	0.89	**1.3**
Sweden [45]	last year	2.19	1.27	**2.4**	0.88	1.01	**1.9**
USA [45]	last year	1.42	1.41	**1.7**	0.69	1.09	**1.6**
Japan [28]	lifetime	1785	13.84	**10.3**	118.5	5.348	**5.1**
Japan [28]	last 3 months	22.45	0.766	**39.3**	1.843	0.437	**10.7**

If the activity of homosexual individuals is similar to that of heterosexual individuals, the contribution of MSM to STI transmission is less than we suspected (as seen in Fig. 4). Under the realistic MSM population estimated from the previous studies shown in Table 1, prevention measures focusing on MSM are not efficient as long as there is not an explosive spread among the MSME population (Eq. (16) does not hold). It should be noted that in this model, MSMW is nearly twice as active as the other groups because it includes both homosexuality and heterosexuality. This seems to be an overestimation, but nevertheless, the impact of MSMW is not necessarily significant in this model. In the case that MSM is not important, it is more efficient to concentrate measures on women than men. Interestingly, a study published by Kahn et al. [53] in 1997 estimated that the annual number of HIV infections in the United States was approximately 40,000 and that infections transmitted by bisexual persons accounted for only 200–600 of those. Therefore, they concluded that transmission via bisexual contact was a relatively minor component of all HIV transmissions in the United States, and it seems that their findings are consistent with our model results [53].

## Conclusion

In the current study, we constructed an STD model in which the population is divided into four subpopulations, women, MSMW, MSME, and MSW, and derived the analytical formula for type-reproduction numbers. As research on actual sexual contacts, including homosexuality, progresses in the future, this formula will be useful for developing preventive strategies. What we can say now is that it is important to simultaneously prevent both homosexual and heterosexual transmission to suppress STIs because MSM and mother-to-child transmission rates do not have a strong synergistic effect. Furthermore, our study is the first to quantify the effects of bisexual bridges on the spread of STIs. Understanding the potential role of MSMW and MSME in STI transmission from MSM to women is epidemiologically important. Our model shows the impact of bisexual bridge on the spread of STIs does not outweigh their population size.

## Supplementary Information

**Additional file 1.** Mathematica code example for calculating the type-reproduction number.

## Data Availability

The authors declare that all data supporting the findings of this study are available within the article.

## Abbreviations

STIs: Sexually transmitted infections
MSW: Men who have sex with women only
MSM: Men who have sex with men
MSME: Men who have sex with men exclusively
MSMW: Men who have sex with men and women
