# Supplementary material for: The effect of men who have sex with men (MSM) on the spread of sexually transmitted infections
Source: Theor Biol Med Model. 2021 Oct 11;18:18. doi: 10.1186/s12976-021-00148-9 (PMC8504019; doi:10.1186/s12976-021-00148-9)
Supplement: Supplementary file 1 — Additional file 1. Mathematica code example for calculating the type-reproduction number. [file 12976_2021_148_MOESM1_ESM.docx]

**Appendix**

Mathematica code example for calculating the type-reproduction number.

(* matrix in Eq. 9 *)

A = {{bw, bw, 0, 0, 0, 0, 0, 0},

{0, 0, RVbw, RHbw, 0, 0, RVmw, RHmw},

{bb, bb, 0, 0, 0, 0, 0, 0},

{RVwb, RHwb, RVbb, RHbb, RVhb, RHhb, 0, 0},

{bh, bh, 0, 0, 0, 0, 0, 0},

{0, 0, RVbh, RHbh, RVhh, RHhh, 0, 0},

{bm, bm, 0, 0, 0, 0, 0, 0},

{RVwm, RHwm, 0, 0, 0, 0, 0, 0}};

MatrixForm[A]

(*basic reproduction number (cannot be calculated analytically)*)

Simplify[Eigenvalues[A]]

(*type-reproduction number of women*)

B1 = {{0, 0, 0, 0, 0, 0, 0, 0},

{0, 0, RVbw, RHbw, 0, 0, RVmw, RHmw},

{0, 0, 0, 0, 0, 0, 0, 0},

{0, 0, 0, 0, 0, 0, 0, 0},

{0, 0, 0, 0, 0, 0, 0, 0},

{0, 0, 0, 0, 0, 0, 0, 0},

{0, 0, 0, 0, 0, 0, 0, 0},

{0, 0, 0, 0, 0, 0, 0, 0}}

Simplify[Eigenvalues[Ta.Inverse[IdentityMatrix[8] - A + B1]]]

(*type-reproduction number of MSMW*)

B2= {{0, 0, 0, 0, 0, 0, 0, 0},

{0, 0, 0, 0, 0, 0, 0, 0},

{0, 0, 0, 0, 0, 0, 0, 0},

{RVwb, RHwb, RVbb, RHbb, RVhb, RHhb, 0, 0},

{0, 0, 0, 0, 0, 0, 0, 0},

{0, 0, 0, 0, 0, 0, 0, 0},

{0, 0, 0, 0, 0, 0, 0, 0},

{0, 0, 0, 0, 0, 0, 0, 0}}

Simplify[Eigenvalues[Ta.Inverse[IdentityMatrix[8] - A + B2]]]

(*type-reproduction number of MSME*)

B3= {{0, 0, 0, 0, 0, 0, 0, 0},

{0, 0, 0, 0, 0, 0, 0, 0},

{0, 0, 0, 0, 0, 0, 0, 0},

{0, 0, 0, 0, 0, 0, 0, 0},

{0, 0, 0, 0, 0, 0, 0, 0},

{0, 0, RVbh, RHbh, RVhh, RHhh, 0, 0},

{0, 0, 0, 0, 0, 0, 0, 0},

{0, 0, 0, 0, 0, 0, 0, 0}}

Simplify[Eigenvalues[Ta.Inverse[IdentityMatrix[8] - A + B3]]]

(*type-reproduction number of MSW*)

B4= {{0, 0, 0, 0, 0, 0, 0, 0},

{0, 0, 0, 0, 0, 0, 0, 0},

{0, 0, 0, 0, 0, 0, 0, 0},

{0, 0, 0, 0, 0, 0, 0, 0},

{0, 0, 0, 0, 0, 0, 0, 0},

{0, 0, 0, 0, 0, 0, 0, 0},

{0, 0, 0, 0, 0, 0, 0, 0},

{RVwm, RHwm, 0, 0, 0, 0, 0, 0}}

Simplify[Eigenvalues[Ta.Inverse[IdentityMatrix[8] - A + B4]]]
